# 428. Assessing the Confidence, Knowledge and Preferences of Hospital Staff with Regards to Personal Protective Equipment (PPE) Practices During the COVID-19 Pandemic

**DOI:** 10.1093/ofid/ofab466.628

**Published:** 2021-12-04

**Authors:** Rachel Brown, Sharon Markman, Amanda Brown, Rukhshan Mian, Vineet Arora, Craig Umscheid

**Affiliations:** UChicago Medicine, Chicago, Illinois

## Abstract

**Background:**

Effective use of personal protective equipment (PPE) by hospital staff is critical to prevent transmission of COVID-19. This study examines hospital staff confidence in and knowledge of effective PPE use, and their preferences for learning about PPE practices.

**Methods:**

Three isolation precautions signs were created for use in the care of those with or under investigation for COVID-19 infection: first, a special respiratory precautions sign designed by infection control; and next, two signs outlining proper donning and doffing practices – one created internally with the support of health literacy, and another developed with a design firm (IDEO) using principles of human-centered design (Figure 1). All signs were used for ≥ 10 weeks prior to distribution of a questionnaire (REDCap) to clinical and non-clinical hospital staff. Those who had not worked on hospital units during the pandemic (after March 15, 2020) were excluded. The 38-item survey was sent by supervisors over email between July 14-31, 2020, and examined demographics, confidence in and knowledge of PPE best practices, and preferences for each precaution sign with regards to trustworthiness, ease of following, informative content, and clarity of image/layout. Responses were reported using descriptive statistics. A non-parametric test of trends compared staff preferences across signs. Logistic regression examined the association between answering all knowledge-based questions correctly and staff role and confidence in PPE practices (Stata).

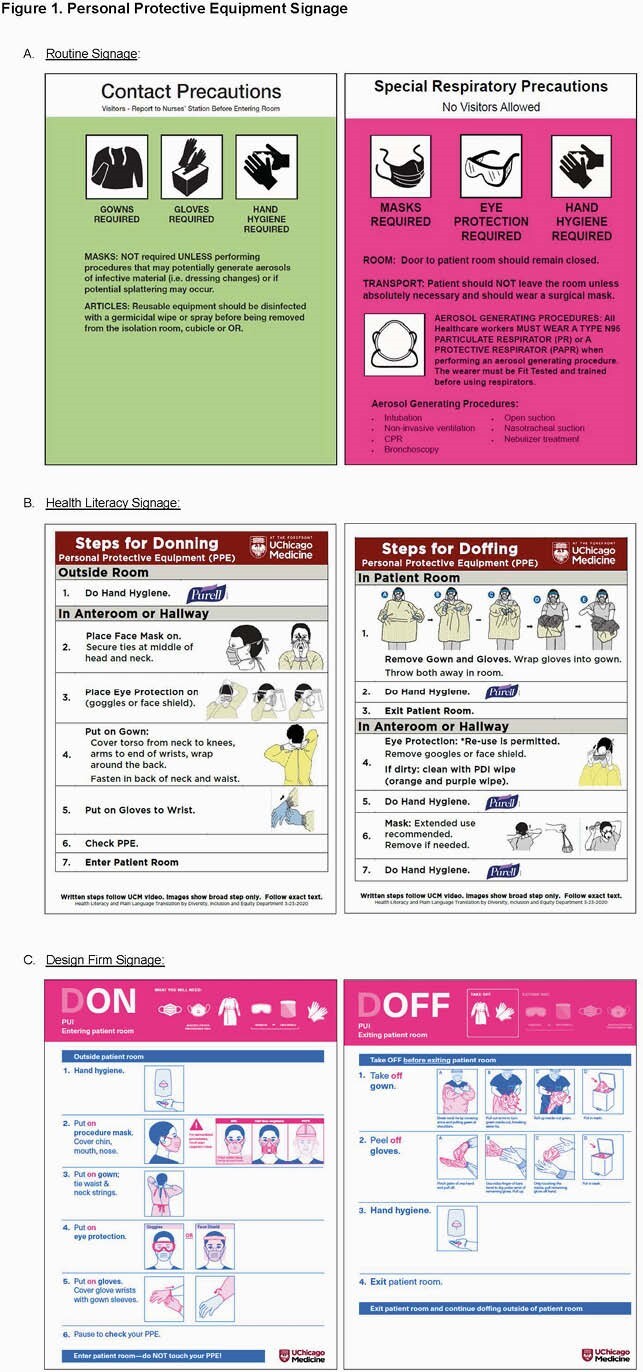

**Results:**

Of the 531 respondents, 461 were eligible for inclusion. The majority were female, white, and not high risk for COVID-19 (Table 1). Most were confident about PPE use, correctly answered questions examining knowledge of PPE best practices, and found PPE signage helpful (Table 2). Staff preferred the professionally designed sign for informative content (p< 0.01) and clear imagery/layout (p=0.01) (Table 3). Confidence in PPE practices and physician or nurse roles were associated with answering all knowledge-based questions correctly (p< 0.001 and p=0.04, respectively).

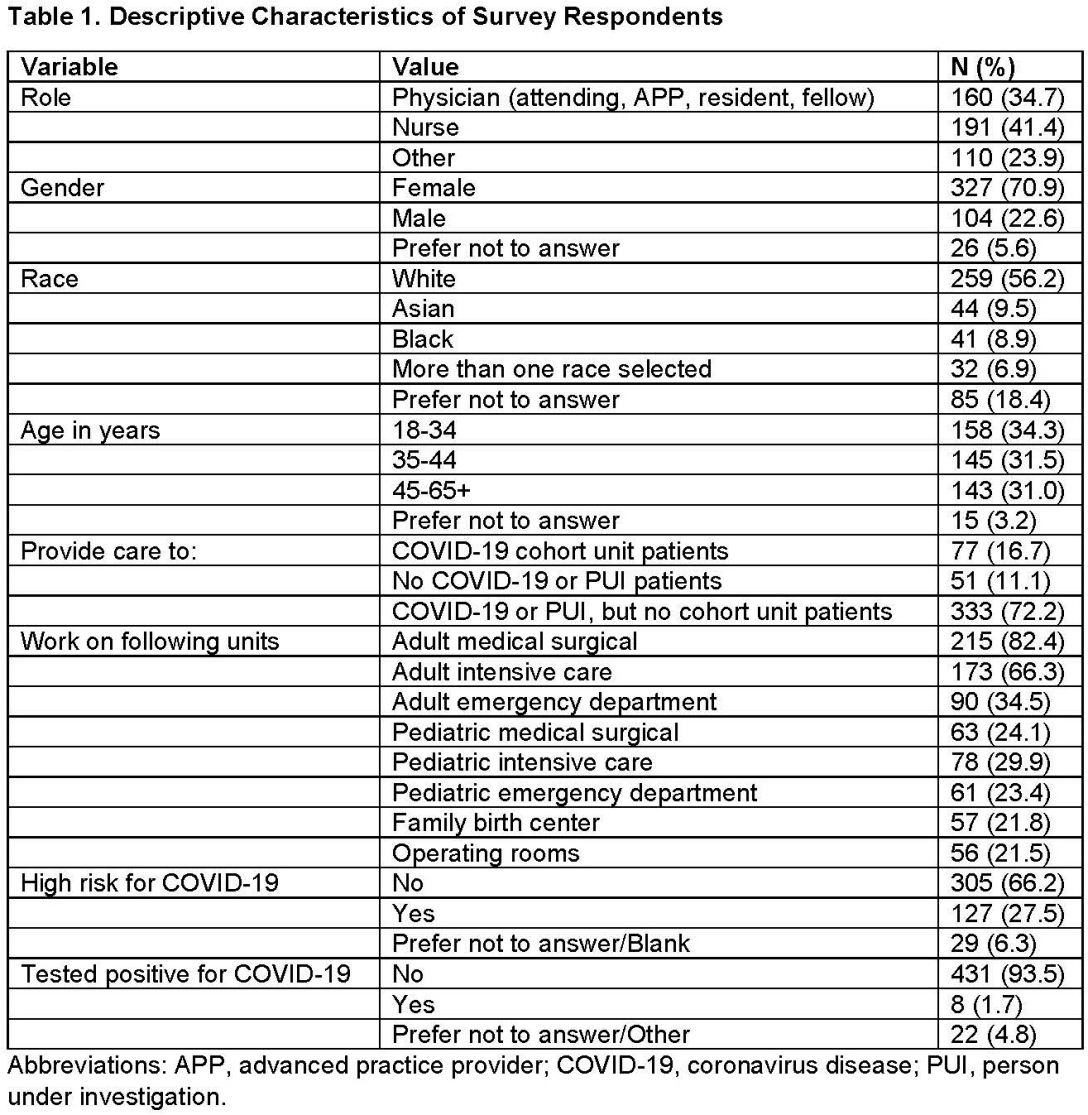

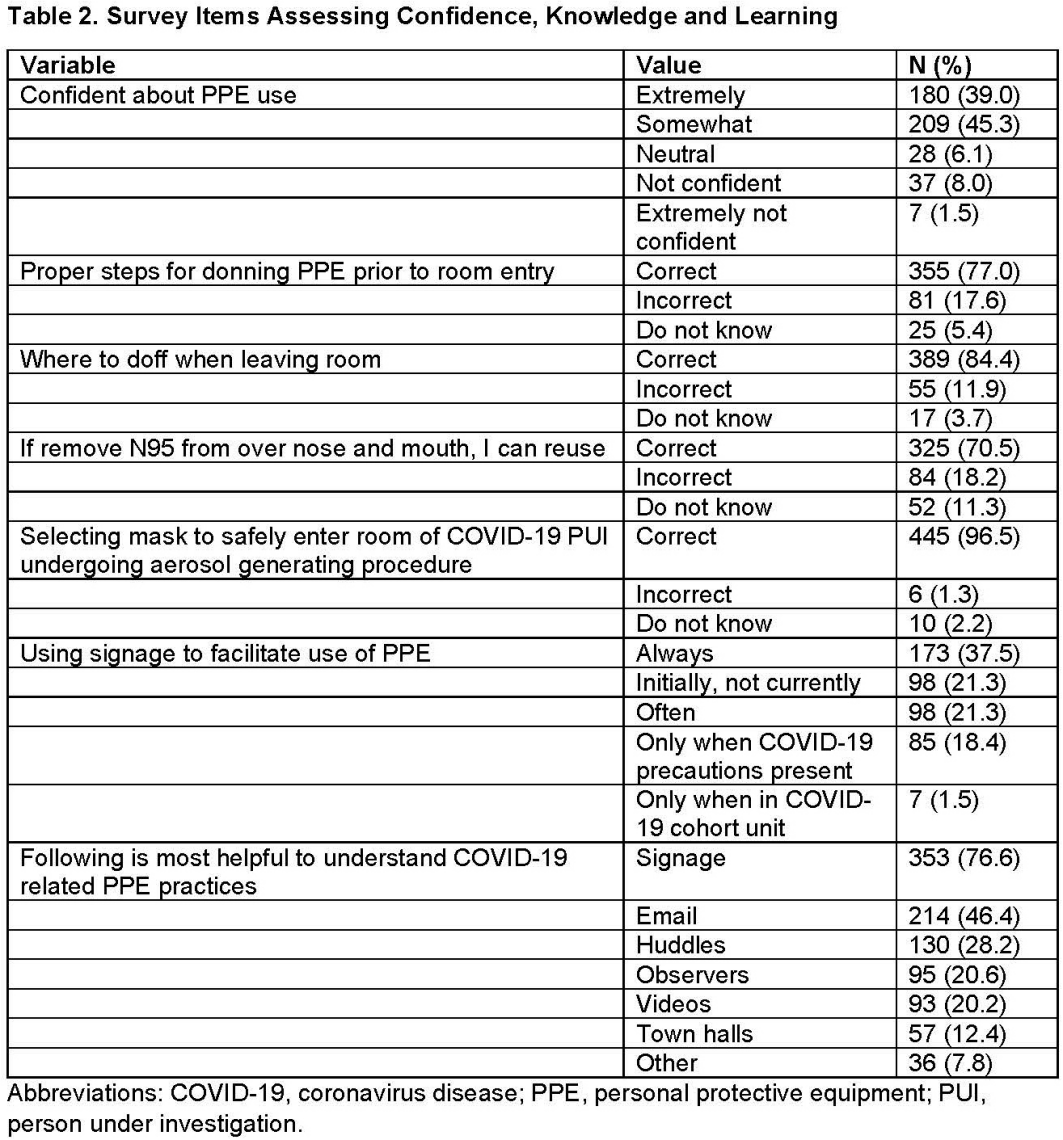

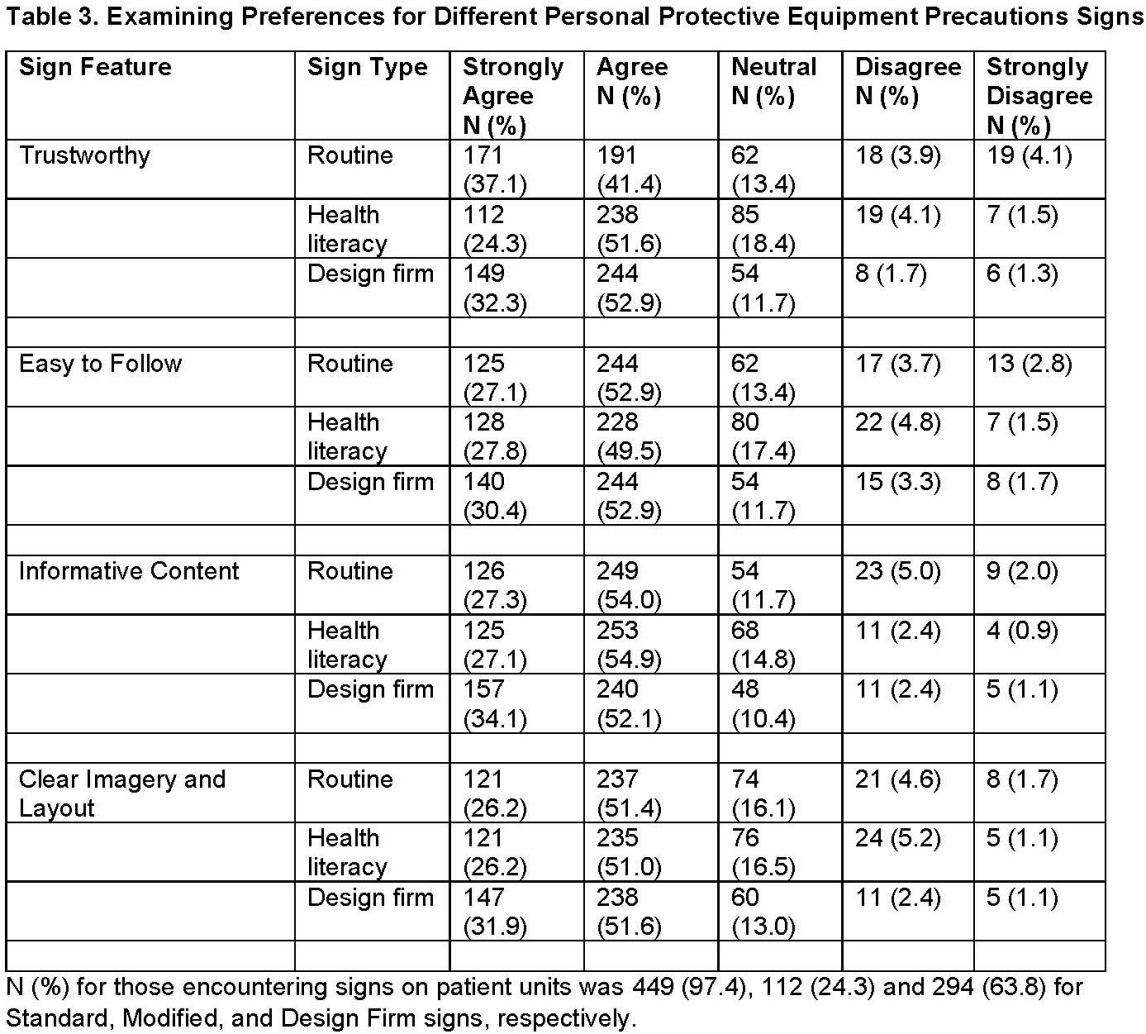

**Conclusion:**

In a convenience sample of hospital staff, most were confident and knowledgeable about PPE use, found PPE signage helpful, and preferred professionally designed signs.

**Disclosures:**

**All Authors**: No reported disclosures

